# The combination of chronic stress and smoke exacerbated depression-like changes and lung cancer factor expression in A/J mice: Involve inflammation and BDNF dysfunction

**DOI:** 10.1371/journal.pone.0277945

**Published:** 2022-11-23

**Authors:** Bai-Ping Liu, Cai Zhang, Yong-Ping Zhang, Kang-Wei Li, Cai Song

**Affiliations:** 1 Research Institute for Marine Drugs and Nutrition, College of Food Science and Technology, Guangdong Ocean University, Zhanjiang, China; 2 Key laboratory of Aquatic Product Processing, Guangdong Ocean University, Zhanjiang, China; 3 Marine Medical Research and Development Centre, Shenzhen Institute of Guangdong Ocean University, Shenzhen, China; Universidade de Sao Paulo, BRAZIL

## Abstract

**Objective:**

Depression is positively correlated with the high incidence and low survival rate of cancers, while more cancer patients suffer depression. However, the interaction between depression and cancer, and possible underline mechanisms are unclear.

**Methods:**

Chronic unpredictable mild stress (CUMS) was used to induce depression, and smoke to induce lung cancer in lung cancer vulnerable AJ mice. After 8 weeks, sucrose preference and forced swimming behaviors were tested. Blood corticosterone concentration, and levels of cytokines, lung cancer-related factors, brain-derived neurotrophic factor (BDNF) and apoptosis-related factors in the lung, amygdala and hippocampus were measured.

**Results:**

Compared to control group, CUMS or smoke decreased sucrose consumption and increased immobility time, which were deteriorated by stress+smoke. CUMS, smoke or both combination decreased mononuclear viability and lung TNF-α concentration, increased serum corticosterone and lung interleukin (IL)-1, IL-2, IL-6, IL-8, IL-10, IL-12 and HSP-90α concentrations. Furthermore, stress+smoke caused more increase in corticosterone and IL-10, but decreased TNF-α. In parallel, in the lung, Bcl-2/Bax and lung cancer-related factors CDK1, CDC20, P38α etc were significantly increased in stress+smoke group. Moreover, CUMS decreased BDNF, while CUMS or smoke increased TrkB and P75 concentrations, which were exacerbated by stress+smoke. In the amygdala, except for CUMS largely increased Bax/Bcl-2 and decreased TrkB, each single factor decreased BDNF and IL-10, but increased P75, IL-1β, IL-12, TNF-α concentrations. Changes in Bax/Bcl-2, IL-10 and TNF-α were further aggravated by the combination. In the hippocampus, except for CUMS largely increased P75 concentration, each single factor significantly increased Bax/Bcl-2 ratio, IL-1β and TNF-α, but decreased BDNF, TrkB and IL-10 concentrations. Changes in Bax, Bax/Bcl-2, IL-10 and TNF-α were further aggravated by the combination.

**Conclusion:**

These results suggest that a synergy between CUMS and smoke exposure could promote the development of depression and lung cancer, through CUMS increased the risk of cancer occurrence, and conversely lung cancer inducer smoke exposure deteriorated depressive symptoms.

## 1. Introduction

Epidemiological surveys showed that patients with cancer are more inclined to depressive symptoms than the normal, while depression was positively correlated with the high incidence of cancers and low survival rates, especially lung cancer [1,2]. Thus, there must be some common factors or synergy between the two diseases. However, very few studies have explored the interaction and possible underline mechanism between depression and lung cancer in both the brain and immune system.

According to macrophage/T-lymphocyte theory of depression, excessive inflammatory response may contribute to depression. First, as a trigger of depression, chronic stress can increase inflammatory response [3]. Second, pro-inflammatory cytokines can induce the hyperactivity of the Hypothalamic-Pituitary-Adrenal (HPA) axis, and release glucocorticoid (GC) through the phospholipase A2 (PLA2)-prostaglandin E2 (PGE2)-corticotropin-releasing factor pathway [4]. Third, pro-inflammatory cytokines can activate indoleamine 2, 3-dioxygenase, which reduces the serotonin availability to the brain via slipping up tryptophan [5]. Indeed, in depressed patients, the concentrations of pro-inflammatory cytokines are increased, such as interleukin (IL)-1β, IL-6, interferon-γ (IFN-γ) and tumor necrosis factor-α (TNF-α), while anti-inflammatory cytokines are decreased, such as IL-10 and IL-4 [6–8].

Chronic stress is considered to be the most significant environmental factor in the etiology of depression [9–12]. Previously, many studies, including from our team, have reported that chronic unpredictable mild stress (CUMS) can induce a valid model for studying the network among the activation of the HPA axis, peripheral and center inflammatory response and glial cell functions in depression [3,13–15].

Many factors may involve in cancer development, such as smoking for lung cancer [16]. However, epidemiologic and clinical studies have found that chronic stress is not only a trigger for depression [10,17], but also an inducer of cancer onset and progression [18–21], especially for lung cancer [2,22]. In the periphery, on the one hand, chronic stress could activate HPA axis and sympathetic nervous system resulting in the release of GC hormones and catecholamine [23]. These changes may induce lymphocyte apoptosis and reduce immune function through activating the glucocorticoid receptor (GR) on immune cells [24–26]. GR can control the activating protein (AP)-1 transcription factor, which regulates the expression of genes involved in lymphocyte growth, differentiation and transformation [27–29], reducing telomerase activity of immune cells and accelerating immune-senescence [30,31]. It is well known that immune suppression or ageing increases the susceptibility to cancers [32,33]. Chronic stress, on the other hand, can promote the formation of blood vessels [18], which may provide the appropriate environment for cancer cell dissemination.

As mentioned above, excessive inflammatory response plays an important role in CUMS-induced depression [34,35]. Recently, the hypothesis of cancer proposed that excessive inflammation can speed cancer progression and decrease patient survival [36]. Tumor-associated inflammation mainly includes tumor necrosis factors, inflammasomes, cytokines, chemokines and transcription factors [37]. Similarly, TNF-α, IL-6 and IL-1β mediate cancer-associated inflammation [38,39], and promote tumorigenesis through generation of a profitable environment by enhancing recruitment of immune-suppressor cells [40]. Therefore, the inflammatory response plays important roles in both depression and tumorigenesis. However, the specific role of chronic stress promotes cancer onset or progression, and conversely cancer induces depression mood or depression-like neuropathological changes are all unknown.

In depressed patients or in the depression model induced by CUMS, the expression of brain-derived neurotrophic factor (BDNF) and its high affinity receptor TrkB (related to neuronal survival) are decreased, while its low affinity receptor P75 (related to neuronal apoptosis) is increased in the limbic system of the brain, which could contribute to neuronal apoptosis [41]. These changes can be attenuated by effective antidepressant treatment [42,43]. Therefore, neurotrophin hypothesis of depression has been raised [44,45]. However, in the periphery, neurotrophins may play opposite roles, such as enhancing tumor proliferation and metastasis. It was reported that BDNF binds to tyrosine kinase B (TrkB) receptor or P75 leads to the activation of PI3K/AKT, RAS/ERK, AMPK/ACC, PLC/PKC and JAK/STAT signalings [46,47], which resulting in tumorigenesis and progression of malignancies, including breast cancer [48], lung cancer [49] and neuroblastoma cancer [50]. As well, many studies have indicated that BDNF/TrkB signaling was strongly associated with tumor progression [51,52]. However, the interaction between depression/stress and lung cancer in the brain and peripheral neurotrophin functions and the implication in cancer developments are also unknown.

In the neuropathology of depression, the amygdala is a critical brain area, which regulates emotion, sensory information and negative stimuli [53]. The hippocampus is the most important brain region controlling emotion and cognition. Increased neuroinflammation and neuronal apoptosis and decreased neurotrophins, such as BDNF system were consistently reported in depressed patients [54]. The disruption of both amygdala and hippocampus functions contributes to the aggravation of patients with major depressive disorder or anxiety [55]. Thus, the present study determined BDNF and its receptors expression, pro- and anti-inflammatory cytokines, as well as apoptosis factors changes in the amygdala and hippocampus.

According to above mentioned unknowns, the present study used the A/J mice, which have successfully been used to develop lung cancer animal model for tobacco smoke carcinogenesis [56], to explore (1) whether CUMS and smoke combination can promote the development of lung cancer; (2) whether a synergistic effect between CUMS and smoke exposure can deteriorate depression-like symptoms and neuropathology; (3) whether the synergistic effects are through common factors, such as excessive corticosterone, inflammation and neurotrophin dysfunction.

## 2. Materials and methods

### 2.1 Animals

Adult male A/J mice (27 g ± 5 g), bred in our laboratory, were housed five per cage. The room temperature was 22 ± 1°C with 12 hours light-dark cycle. Food and water were available *ad libitum*. The experimental protocol was approved by the Bioethics Committee of Guangdong Ocean University, China (Guangdong Ocean University, China, SYXK (Yue) 2014–0053, IACUC- 20180510–013) according to the instruction of the National Institutes of Health Guide for the Care and Use of the Laboratory Animals.

### 2.2 Experimental procedure

A/J mice were randomly divided into four groups as 1) Control group; 2) Stress group; 3) Smoke group; 4) Stress + Smoke group.

#### 2.2.1 CUMS procedure

Mice were subjected to CUMS for 8 weeks as previously established protocols [57,58] with minor modification. The protocol consisted of a variety of stressors as following: food and water deprivation for overnight, overnight illumination, forced swimming at 18°C for 10 min, shaking at 200 rpm for 2 h, restraint for 2 h, cold exposure (4°C) for 1 h, cage tilting at 45° for overnight, soiled cage overnight, in crowded space for overnight, stroboscopic illumination for overnight, light on and off every 3 h for 24 h, isolation overnight, odor overnight. Mice in stress groups suffered from two kinds of arbitrary stressors daily (S1 Table).

#### 2.2.2 Smoke exposure procedure

The mice in the smoke group were placed in a device, which was a rectangular plastic box (60*20*40 cm) with a lid that can be opened and with an exhaust fan on top to allow air to circulate (S1 Fig). The mice were exposed to tobacco (Jiangmen Xinhui District Tobacco Factory Co. LTD) twice a day at dose 4 g each time for 8 weeks. The smoking duration was 15 min with 15 min interval [59].

### 2.3 Sucrose preference test (SPT)

The SPT was performed as previously published protocols [57]. Mice were trained to adapt to the sucrose solution (1%, w/v) in a group before test. On day 1, mice were offered two bottles 1% sucrose water to consume. On day 2, mice were offered one bottle 1% sugar water and one bottle fresh water. On day 3, mice were individually housed in the cage with food and water deprivation for 24 h. On day 4, each mouse was offered one bottle pre-weighed 1% sugar water and one bottle pre-weighed fresh water. Then the consumption of sugar water and fresh water were weighed after 4 h. The sucrose preference (SP) was calculated based on the formula: SP (%) = sucrose intake/ (sucrose intake + water intake) × 100%.

### 2.4 Forced swimming test (FST)

The FST was conducted as previously published protocols [60]. The apparatus consisted of a plastic cylinder (11 cm diameter × 30 cm height) containing 20 cm deep water at 25°C ± 1°C. On the first day, pretest was conducted for 10 min. After 24 h, test was performed 5 min, and the duration of immobility time was recorded in 5 min.

### 2.5 Collection of serum, amygdala, hippocampus and lung samples

Serum samples were collected immediately after mice were decapitated. Then the lungs and amygdala were quickly separated from mice on ice. The right lung was fixed in neutral 10% formalin for histological staining. The left lung, whole amygdala and hippocampus were flash frozen in liquid nitrogen and stored at -80°C for subsequent ELISA, q-PCR and western blot assays.

### 2.6 Histologic staining

After fixing for 24 hours, the right lung was embedded in paraffin, and cut into 4-μm-thick sections, then stained with hematoxylin and eosin (H&E) kit (Beyotime) according to the manufacturer’s instruction.

### 2.7 The proliferation of spleen mononuclear

After decapitation, mononuclear cells were separated from the spleen according to the manufacturer’s protocol for mouse spleen lymphocytes obtained from Tianjin Hao Yang Biotechnology Co., Ltd (Tianjin, China) with minor modification. 4.0×10^5^ cells/well were seeded into 96-well plates. Then cells were stimulated by 20 μg/mL lipopolysaccharide or 10 μg/mL concanavalin A (final concentration). Cells viability was tested by MTT (Solarbio) method after incubating 24 or 48 h at 37°C in a humidified 5% CO_2_–air mixture.

### 2.8 Quantitative reverse transcription-PCR

The total mRNA was extracted from the left lung and amygdala using theTrizol reagents (Invitrogen) according to the manufacturer’s protocol. cDNA was synthesized from 2 μg total RNA using RT kit (Promega). PCR amplifications were performed using SYBR Green Master Mix kit (Takara). The primer sequences and the internal control, β-actin, are listed in Table 1. Fold changes were normalized to β-actin using the 2^-ΔΔCt^- method.

**Table 1:** Primer sequences of quantitative PCR.

Gene name	Primer sequences (5’- 3’)
Bax	F:CGAGTGTCTCCGGCGAATTG
	R:ATGGTGAGCGAGGCGGTGAG
Bcl-2	F:GGTACCGGAGAGCGTTCAGT
	R:CTGCTGCATTGTTCCCGTAG
BDNF	F:AGCTGAGCGTGTGTGACAGT
	R:TCAGTTGGCCTTTGGATACC
TrkB	F:CACACACAGGGCTCCTTA
	R:GTCAGCTCAAGCCAGACACA
P75	F:CCGATGCTCCTATGGCTACT
	R:CTCTGGGCACTCTTCACACA
CDK1	F:GGCAGTTCATGGATTCTTCACTC R:GCCAGTTTGATTGTTCCTTTGTC
CDC20	F:AACAGGAGGAGGAACCAGTGACC
	R:GCACATCCACAGCACTCAGACAG
P38α	F:CTGGACAAGGCGTGTGAAGGC
	R:GCACCATGAGCACCTGGAGAATC
ROS1	F:TGACACGATGCCAGTTGCCTTG
	R:TGTCTGCCTGTGGTTGCTTCTTG
CUEDC	F:GGTGCGAAGCCTGCCATGTC
	R:CTTGCGGAAGCGTGGTGTGG
Gankyrin	F:CGGCTGCACTCCACTCCATTATG
	R:GGCTAAGTGTCCTTGGCTGCTG
β-actin	F:CATCCGTAAAGACCTCTATGCCAAC
	R:ATGGAGCCACCGATCCACA

### 2.9 Western blot

Protein expression of TrkB and P75 was measured by western blot in the amygdala. The total protein was extracted from the amygdala and the concentration was measured with the BCA protein quantitative kit (Dingguo, Beijing) according to the manufacturer’s protocol. 30 μg total protein were separated by 10% SDS PAGE gels and transferred onto PVDF membranes (Millipore, USA). After blocking with 5% non-fat milk for 2 h at room temperature, membranes were incubated with primary and secondary (MultiSciences, 1:5000) antibodies. The primary antibodies include TrkB (1:1000, ab187041, Abcom, UAS), P75 (1:400, sc-271708, Santa Cruz, USA) and β-Actin (1:400, sc-8432, Santa Cruz, USA). The proteins on the membranes were detected by enhanced chemiluminescence (ECL) (Millipore Corp., USA). The bands were analyzed by chemiluminescence system (Tanon 5200, Shanghai, China). All target proteins were quantified by normalizing them to β-Actin re-probed on the same membrane and calculated as a percentage of the control group.

### 2.10 Enzyme-linked immunosorbent assay (ELISA)

The concentration of corticosterone in the serum, lung cancer-related factors CDK1, CDC20 and HSP-90α, TrkB and P75 in the left lung, as well as pro- and anti-inflammatory cytokines IL-1, IL-2, IL-6, IL-8, IL-10, IL-12 and TNF-α, apoptosis-related factors bax and bcl-2 and BDNF both in the left lung, amygdala and hippocampus were determined by ELISA with commercial reagent kits, purchased from Jiangsu Yutong Biotechnology co. Ltd, according to the manufacturer’s instruction.

### 2.11 Statistical analysis

Results are expressed as mean ± standard error of the mean (SEM). Statistical analysis was performed by IBM SPSS Statistics 19.0 software. Data were analyzed by a two-way ANOVA except for GC, mononuclear viability and cancer related factors, which were analyzed by student’s t test. Differences between groups were assessed by a Fisher’s least significant difference (LSD) *post hoc* test. Significance was set at *P* < 0.05.

## 3. Results

### 3.1 CUMS or smoke induced depression-like behaviors, which were deteriorated in the combination group

**Fig 1:**
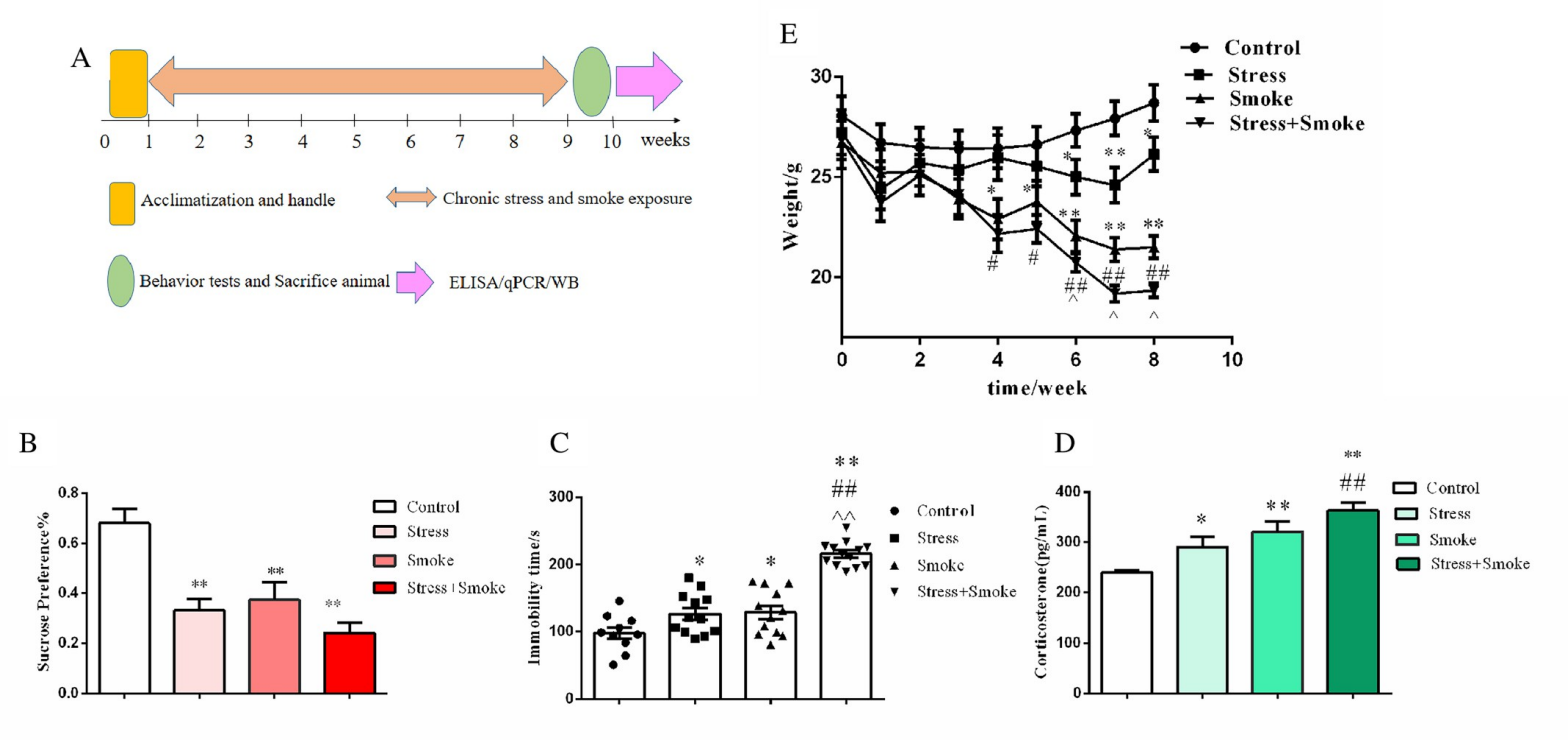
CUMS or smoke induced depression-like behaviors, which were further deteriorated in their combination group. (A) Experiment scheme and timeline. (B) The behavior of sucrose preference was tested after CUMS and/or smoking. (C) The behavior of forced swimming was tested after CUMS and/or smoking. (D) Corticosterone concentration was measured by ELISA kit. (E) The body weight of mice was recorded weekly. The data are expressed as mean ± SEM (n = 11). *P < 0.05, **P < 0.01 versus control group; ^#^*P* < 0.05, ^##^*P* < 0.01 versus stress group; ^^^*P* < 0.05, ^^^^*P* < 0.01 versus smoke group.

The experiment scheme and timeline were shown in Fig 1A. Two-way ANOVA indicated a significant effect of CUMS (SPT: F_1,40_ = 31.991, *P* < 0.01; FST: F_1,40_ = 23.317, *P* < 0.01), smoke (SPT: F_1,40_ = 17.963, *P* < 0.01; FST: F_1,40_ = 25.655, *P* < 0.01) and the interaction between CUMS and smoke (SPT: F_1,40_ = 9.536, *P* < 0.01; FST: F_1,40_ = 4.010, *P* < 0.05) on anhedonia behavior in the SPT and the immobility time in the FST. The *post hoc* test showed that CUMS or smoke markedly decreased sucrose intake in the SPT (*P* < 0.01), while increased immobility time in the FST when compared to control group (stress or smoke, *P* < 0.05). Moreover, the mice in stress+smoke group showed more increase in the immobility time in the FST (*P* < 0.01 versus CUMS or smoke) (Fig 1B and 1C).

In parallel, serum corticosterone concentration was increased compared to control group after CUMS or smoke exposure (stress, *P* < 0.05; smoke, *P* < 0.01). While, this hormone was increased more in stress+smoke group when compared to control or CUMS group (*P* < 0.01) (Fig 1D).

With regards to the gain of body weight, repeated measure ANOVA indicated a significant time effect (F_8, 26_ = 72.528, *P* < 0.01) and an obvious interaction between time and group (F_24, 76_ = 10.288, *P* < 0.01) on the body weight. The *post hoc* test showed that the body weight was significantly decreased compared to control group (both *P* < 0.05) after CUMS or smoke exposure. However, the mice in stress+smoke group lost morebody weights when compared to either CUMS or smoke group (stress, *P* < 0.01; smoke, *P* < 0.05) (Fig 1E).

### 3.2 CUMS enhanced pro-apoptotic and neuroinflammatory factors, induced BDNF dysfunction, which were exacerbated by the combination in the amygdala

**Fig 2:**
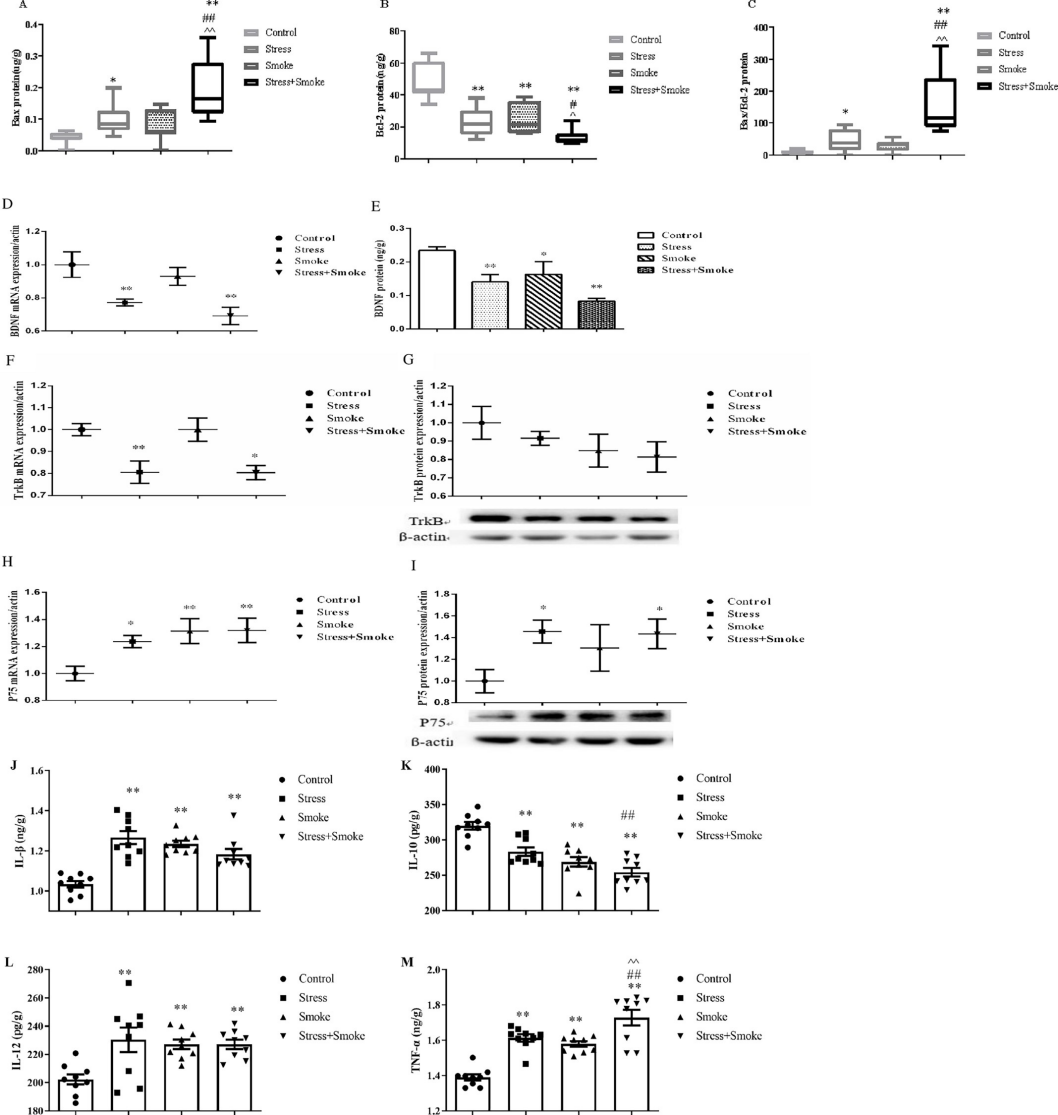
CUMS increased the expression of pro-apoptosis factor, but decreased the expression of anti-apoptosis factor, while the combination of CUMS and smoke exacerbated these changes by increasing inflammation and dysfunction of BDNF system in the amygdala. The concentrations of apoptosis-related factors, neuroinflammatory factors and BDNF by ELISA kits, BDNF, TrkB and P75 mRNA by qPCR, TrkB and P75 protein expression by WB in the amygdala of the mice. (A) Bax protein concentration. (B) Bcl-2 protein concentration. (C) Bax/Bcl-2 ratio. (D) BDNF mRNA expression. (E) BDNF protein concentration. (F) TrkB mRNA expression. (G) TrkB protein expression. (H) P75 mRNA expression. (I) P75 protein expression. (J, K, L and M) The concentrations of IL-1β, IL-10, IL-12 and TNF-α. The data are expressed as mean ± SEM (n = 6–10). *P < 0.05, **P < 0.01 versus control group; ^#^*P* < 0.05, ^##^*P* < 0.01 versus stress group; ^^^*P* < 0.05, ^^^^*P* < 0.01 versus smoke group.

In the amygdala, two-way ANOVA indicated that either CUMS or smoke significantly affected the expression of Bax (stress, F_1,31_ = 25.393, *P* < 0.01; smoke, F_1,31_ = 18.364, *P* < 0.01), Bcl-2 (stress, F_1,30_ = 36.355, *P* < 0.01; smoke, F_1,30_ = 30.182, *P* < 0.01) and Bax/Bcl-2 (stress, F_1,30_ = 20.862, *P* < 0.01; smoke, F_1,30_ = 14.844, *P* < 0.01). Moreover, the interaction between CUMS and smoke exerted a significant effect on the Bax (F_1,31_ = 4.231, *P* < 0.05), Bcl-2 (F_1,30_ = 5.664, *P* < 0.05) and Bax/Bcl-2 (F_1,30_ = 5.724, *P* < 0.01). The *post hoc* test showed that CUMS or the combination significantly increased Bax (stress, *P* < 0.05; combination, *P* < 0.01) and Bax/Bcl-2 (stress, *P* < 0.05; combination, *P* < 0.01), while CUMS, smoke or their combination significantly decreased Bcl-2 (all in *P* < 0.01) when compared to the control group. Again, the increase in Bax and Bax/Bcl-2, and decrease in Bcl-2 were more pronounced in the stress+smoke group when compared to other two factor alone (Bax and Bax/Bcl-2, *P* < 0.01; Bcl-2, *P* < 0.05) (Fig 2A–2C). Moreover, the change of caspase3 mRNA expression is similar to that of Bax/Bcl-2 (S2 Fig).

At the molecular level, two-way ANOVA indicated that CUMS significantly affected BDNF mRNA expression (F_1,31_ = 17.840, *P* < 0.01) and protein concentration (F_1,30_ = 15.810, *P* < 0.01), TrkB mRNA expression (F_1,31_ = 22.224, *P* < 0.01), P75 mRNA (F_1,31_ = 3.956, *P* ≤ 0.05) and protein expression (F_1,19_ = 10.393, *P* < 0.01). Smoke exposure significantly affected P75 mRNA expression (F_1,31_ = 7.800, *P* < 0.01) and BDNF protein concentration (F_1,30_ = 8.580, *P* < 0.01). The *post hoc* test showed that CUMS or the combination significantly decreased mRNA expression of BDNF (all in *P* < 0.01) and TrkB (CUMS, *P* < 0.01; combination, *P* < 0.05), while increased P75 (CUMS, *P* < 0.05; combination, *P* < 0.01). Smoke exposure only increased P75 expression (*P* < 0.01). Similar to mRNA results, at the protein level, CUMS and the combination largely decreased the concentration of BDNF (all in *P* < 0.01), while increased P75 expression (all in *P* < 0.05). Smoke partially, but significantly decreased BDNF concentration (*P* < 0.05) (Fig 2D–2I).

With regards to cytokines change, two-way ANOVA indicated that CUMS or smoke significantly affected IL-1β (CUMS, F_1,32_ = 6.233, *P* < 0.05; smoke, F_1,32_ = 14.54, *P* < 0.01), IL-10 (CUMS, F_1,32_ = 42.98, *P* < 0.01; smoke, F_1,32_ = 17.62, *P* < 0.01), IL-12 (CUMS, F_1,32_ = 4.204, *P* < 0.05; smoke, F_1,32_ = 7.131, *P* < 0.05) and TNF-α (CUMS, F_1,32_ = 31.24, *P* < 0.01; smoke, F_1,32_ = 46.88, *P* < 0.01) concentrations. Moreover, the interaction between CUMS and smoke also significantly affected the concentrations of IL-1β (F_1,32_ = 35.81, *P* < 0.01), IL-10 (F_1,32_ = 3.307, *P* = 0.0783) and IL-12 (F_1,32_ = 7.128, *P* < 0.05). The *post hoc* test showed that CUMS, smoke or their combination significantly increased the concentrations of IL-1β, IL-12 and TNF-α (all in *P* < 0.01), while decreased IL-10 (all in *P* < 0.01). Again, the changes in IL-10 and TNF-α were exacerbated in the combination group when compared to CUMS or smoke alone (all in *P* < 0.01) (Fig 2J–2M).

### 3.3 CUMS increased pro-apoptotic factors and proinflammatory cytokines, induced BDNF dysfunction, which were exacerbated by the combination in the hippocampus

**Fig 3:**
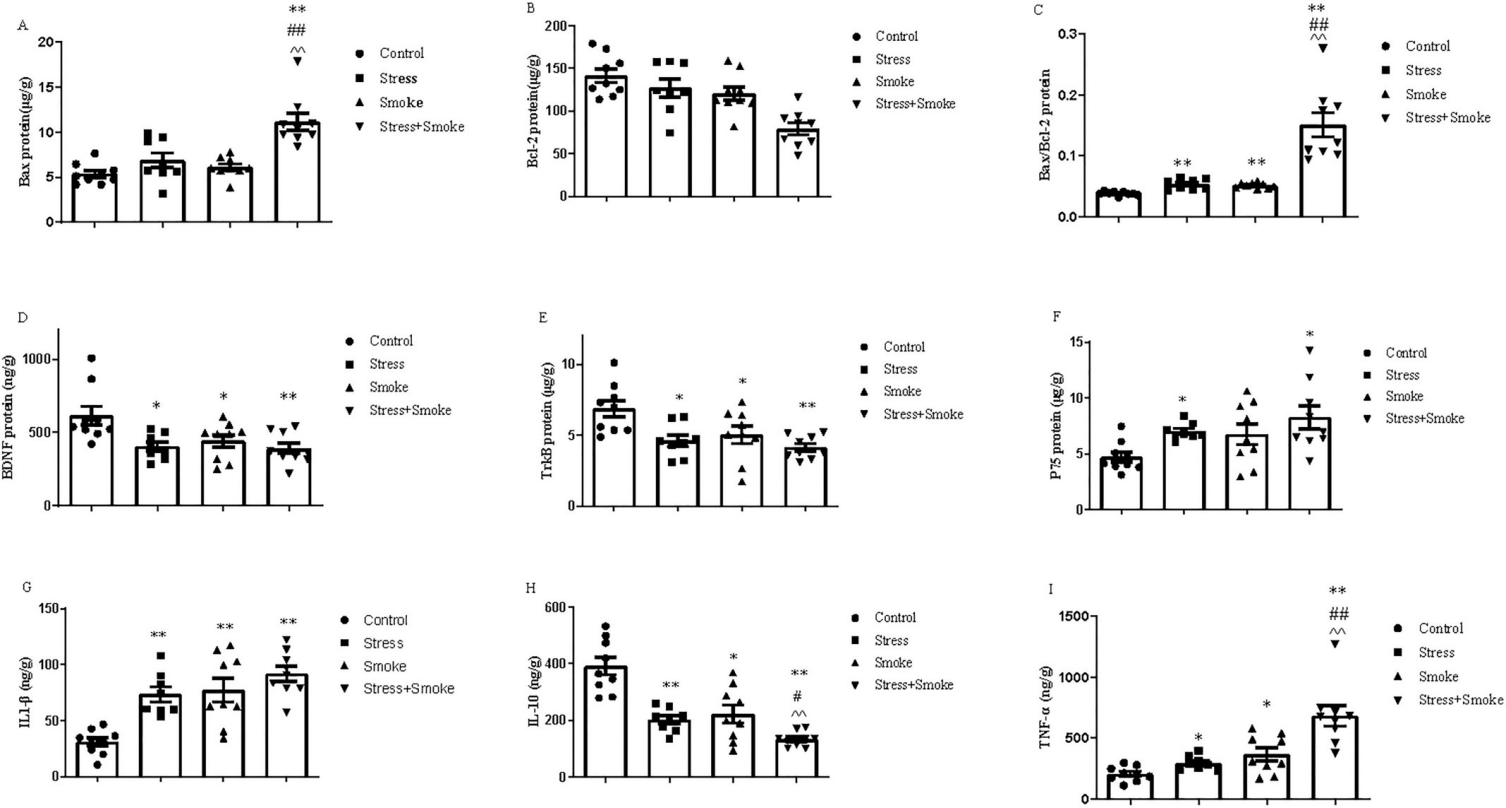
CUMS increased the concentration of pro-apoptosis factor, while the combination of CUMS and smoke exacerbated these changes by increasing inflammation and dysfunction of BDNF system in the hippocampus. The concentrations of apoptosis-related factors, neuroinflammatory factors, BDNF, TrkB and P75 by ELISA kits in the hippocampus of the mice. (A, B and C) The concentrations of Bax and Bcl-2, and Bax/Bcl-2 ratio. (D, E and F) The concentrations of BDNF, TrkB and P75. (G, H and I) The concentrations of IL-1β, IL-10 and TNF-α. The data are expressed as mean ± SEM (n = 8–9). *P < 0.05, **P < 0.01 versus control group; ^#^*P* < 0.05, ^##^*P* < 0.01 versus stress group; ^^^*P* < 0.05, ^^^^*P* < 0.01 versus smoke group.

In the hippocampus, two-way ANOVA indicated that either CUMS or smoke significantly affected the concentrations of Bax (stress, F_1,31_ = 13.99, *P* < 0.01; smoke, F_1,31_ = 23.84, *P* < 0.01), Bcl-2 (stress, F_1,31_ = 17.09, *P* < 0.01; smoke, F_1,31_ = 11.14, *P* < 0.01) and Bax/Bcl-2 (stress, F_1,31_ = 27.98, *P* < 0.01; smoke, F_1,31_ = 30.25, *P* < 0.01). Moreover, the interaction between CUMS and smoke exerted a significant effect on the Bax (F_1,31_ = 6.963, *P* < 0.05) and Bax/Bcl-2 (F_1,31_ = 16.40, *P* < 0.01). The *post hoc* test showed that CUMS or smoke alone significantly increased Bax/Bcl-2 (*P* < 0.01) when compared to the control group. Moreover, the combination of CUMS and smoke caused more significantly increased concentrations of Bax (*P* < 0.01) and Bax/Bcl-2 (*P* < 0.01) when compared to each single group (Fig 3A–3C).

At the molecular level, two-way ANOVA indicated that CUMS or smoke significantly affected the concentrations of BDNF (stress: F_1,31_ = 4.130, *P* ≤ 0.05; smoke: F_1,31_ = 8.042, *P* < 0.01), TrkB (stress: F_1,31_ = 5.450, *P* < 0.05; smoke: F_1,31_ = 10.11, *P* < 0.01) and P75 (stress: F_1,31_ = 4.649, *P* < 0.05; smoke: F_1,31_ = 6.191, *P* < 0.05). The *post hoc* test showed that CUMS, smoke or their combination significantly decreased the concentrations of BDNF (stress: *P* < 0.05; smoke: *P* < 0.05; combination: *P* < 0.01) and TrkB (stress: *P* < 0.05; smoke: *P* < 0.05; combination: *P* < 0.01) when compared to the control group, while CUMS or the combination increased P75 (*P* < 0.05) (Fig 3D–3F).

With regards to cytokines change, two-way ANOVA indicated that CUMS or smoke significantly affected IL-1β (CUMS, F_1,31_ = 19.39, *P* < 0.01; smoke, F_1,31_ = 15.23, *P* < 0.01), IL-10 (CUMS, F_1,31_ = 23.76, *P* < 0.01; smoke, F_1,31_ = 32.04, *P* < 0.01) and TNF-α (CUMS, F_1,31_ = 27.10, *P* < 0.01; smoke, F_1,31_ = 14.74, *P* < 0.01) concentrations. Moreover, the interaction between CUMS and smoke also significantly affected the concentrations of IL-10 (F_1,31_ = 4.208, *P* < 0.05) and TNF-α (F_1,31_ = 4.709, *P* < 0.05). The *post hoc* test showed that CUMS, smoke or their combination significantly increased the concentrations of IL-1β (*P* < 0.01) and TNF-α (CUMS: *P* < 0.05; smoke: *P* < 0.05; combination: *P* < 0.01), while decreased IL-10 (CUMS: *P* < 0.01; smoke: *P* < 0.05; combination: *P* < 0.01) when compared to the control group. Again, the changes in IL-10 (CUMS: *P* < 0.05; smoke: *P* < 0.01;) and TNF-α (*P* < 0.01) were exacerbated in the combination group when compared to CUMS or smoke alone (Fig 2G–2I).

### 3.4 The combination of CUMS and smoke enhanced the expression of lung cancer-related factors in the lung

**Fig 4:**
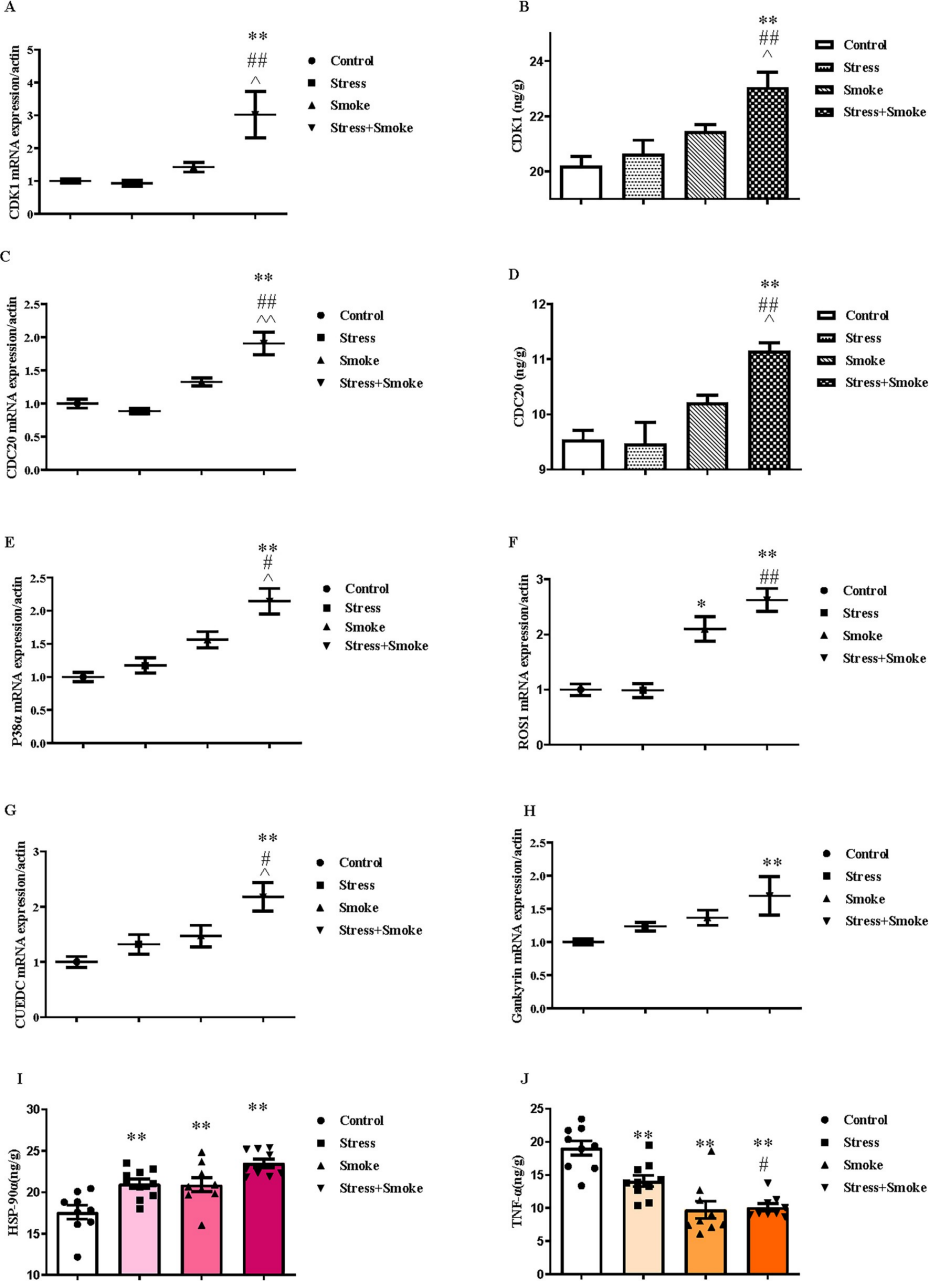
The combination of CUMS and smoke exacerbated the changes in the lung cancer-related factors in the left lung. The mRNA expression of lung cancer-related factors in the lung were measured by qPCR, while the concentrations were detected by ELISA kits. (A) CDK1 mRNA expression. (B) CDK1 protein concentration. (C) CDC20 mRNA expression. (D) CDC20 protein concentration. (E, F, G and H) The mRNA expression of P38α, ROS1, CUEDC and Gankyrin. (I and J) The protein concentrations of HSP-90α and TNF-α. The data are expressed as mean ± SEM (n = 9–10). **P < 0.01 versus control group; ^#^*P* < 0.05, ^##^*P* < 0.01 versus stress group; ^^^*P* < 0.05, ^^^^*P* < 0.01 versus smoke group.

After H&E staining the right lung, no significant changes in the cancer size were found in CUMS, smoke or their combination group (S3 Fig). However, at mRNA level, for lung cancer related factor ROS1, CUMS alone did not induce any significant change in ROS1. However, smoke alone significantly increased ROS1 expression (*P* < 0.01). The combination of CUMS and smoke also induced more significant increase in ROS1 (*P* < 0.01) than it in the control or CUMS group, but no markedly difference from the smoke group. Similar to other pathological changes, the combination of CUMS and smoke caused the most pronounced increase in CDK1 (versus control, *P* < 0.01; stress, *P* < 0.01; smoke, *P* < 0.05), CDC20 (*P* < 0.01), P38α (versus control, *P* < 0.01; stress, *P* < 0.01; smoke, *P* < 0.05) and CUEDC (versus control, *P* < 0.01; stress, *P* < 0.05; smoke, *P* < 0.05) even though these factors were not significant changed in the single factor group. Moreover, the expression of Gankyrin in the combined group was significantly increased when compared to the control group (*P* < 0.01), but no difference to other groups. At the protein level, CUMS or smoke alone did not change the expression of lung cancer-related factors. However, the combination significantly increased protein expression of CDK1 (control, *P* < 0.01; stress, *P* < 0.01; smoke, *P* < 0.05) and CDC20 (control, *P* < 0.01; stress, *P* < 0.01; smoke, *P* < 0.05) when compared to control, CUMS or smoke group (Fig 4A–4H).

Even more important, two-way ANOVA indicated that either CUMS or smoke exposure significantly affected the concentration of HSP-90α (stress, F_1,33_ = 16.774, *P* < 0.01; smoke, F_1,33_ = 15.322, *P* < 0.01) and TNF-α (stress, F_1,33_ = 5.452, *P* < 0.05; smoke, F_1,33_ = 45.725, *P* < 0.01). Moreover, the interaction between CUMS and smoke had a significant effect on TNF-α concentration (F_1,33_ = 7.400, *P* < 0.01), while the change in HSP-90α concentration (F_1,33_ = 3.600, *P* = 0.06) tended to be significant. The *post hoc* test showed that CUMS, smoke or both combination significantly increased HSP-90α concentration (*P* < 0.01) compared to the control group in the left lung, which increase was near statistical significant in stress+smoke group when compared to the changes in stress or smoke group alone. Furthermore, CUMS, smoke or both combination markedly decreased TNF-α concentration (*P* < 0.01) when compared to the control. The lowest TNF-α concentration was found in stress+smoke group than stress alone group (*P* < 0.01) (Fig 4I and 4J).

### 3.5 CUMS, smoke or their combination changed immune function in the left lung

**Fig 5:**
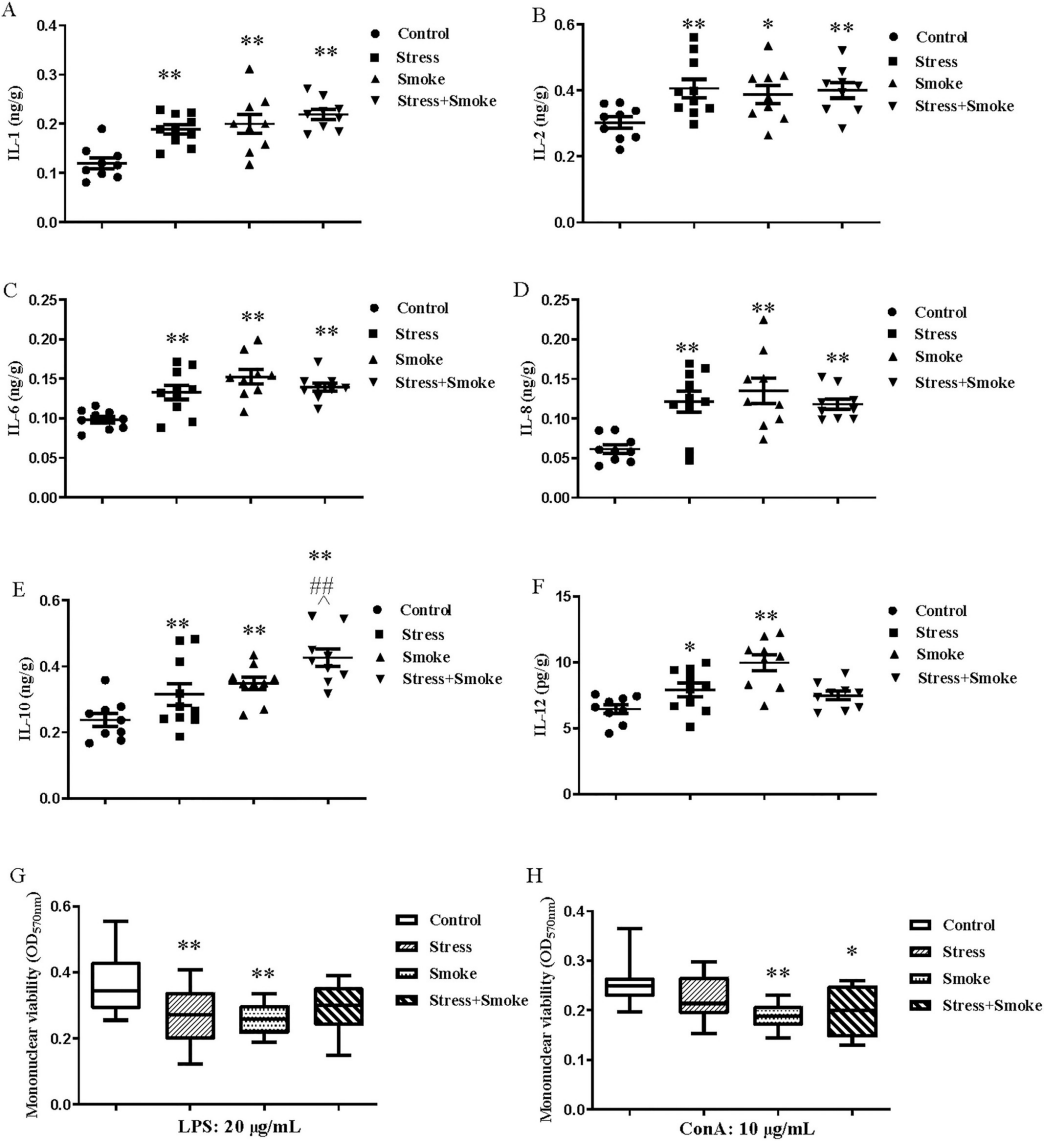
CUMS, smoke or their combination increased pro-inflammatory cytokines production, but decreased peripheral mononuclear cells viability after stimulated by LPS or ConA. The concentration of inflammatory factors in the lung were detected by ELISA kits, while the spleen mononuclear viability was measured by MTT method. (A-F) The concentrations of IL-1, IL-2, IL-6, IL-8, IL-10 and IL-12. (G and H) The viability of peripheral mononuclear cells. The data are expressed as mean ± SEM (n = 9–10) **P < 0.01 versus control group; ^##^*P* < 0.01 versus stress group; ^^^*P* < 0.05 versus smoke group.

Two-way ANOVA indicated that CUMS significantly affected the concentration of IL-1 (F_1,33_ = 11.158, *P* < 0.01), IL-10 (F_1,33_ = 8.802, *P* < 0.01) and IL-12 (F_1,33_ = 5.481, *P* < 0.05). Smoke significantly influenced the concentration of IL-1 (F_1,33_ = 17.409, *P* < 0.01), IL-6 (F_1,33_ = 16.950, *P* < 0.01), IL-8 (F_1,33_ = 9.497, *P* < 0.01), IL-10 (F_1,33_ = 18.124, *P* < 0.01) and IL-12 (F_1,33_ = 10.784, *P* < 0.01). The interaction between CUMS and smoke significantly affected the concentration of IL-6 (F_1,33_ = 10.395, *P* < 0.01), IL-8 (F_1,33_ = 11.473, *P* < 0.01) and IL-12 (F_1,33_ = 17.401, *P* < 0.01), while the concentration of IL-1 (F_1,33_ = 3.458, *P* = 0.07) and IL-2 (F_1,33_ = 3.400, *P* = 0.07) tended to be significant. The *post hoc* test showed that CUMS, smoke or both combination significantly increased the concentration of IL-1 (*P* < 0.01), IL-6 (*P* < 0.01), IL-8 (*P* < 0.01), IL-10 (*P* < 0.01), IL-2 (stress, *P* < 0.01; smoke, *P* < 0.05; combination, *P* < 0.01) and IL-12 (stress, *P* < 0.05; smoke, *P* < 0.01) when compared to the control group. Furthermore, in the combination group, the increase in IL-10 (stress, *P* < 0.01; smoke, *P* < 0.05) were significant compared to the CUMS or smoke alone group (Fig 5A–5F).

With regard to the mononuclear viability, after 20 μg/mL LPS stimulation, mononuclear viability was significantly decreased either in stress group or smoke group compared to the control group (*P* < 0.01). However, the reduction was not significant in the combination group (*P* = 0.067). Upon 10 μg/mL Con A stimulation, largely decreased mononuclear viability was found in smoke or the combination compared to the control group (*P* < 0.01) (Fig 5G and 5H).

### 3.6 CUMS or smoke caused BDNF system dysfunction, which was exacerbated by their combination in the left lung

**Fig 6:**
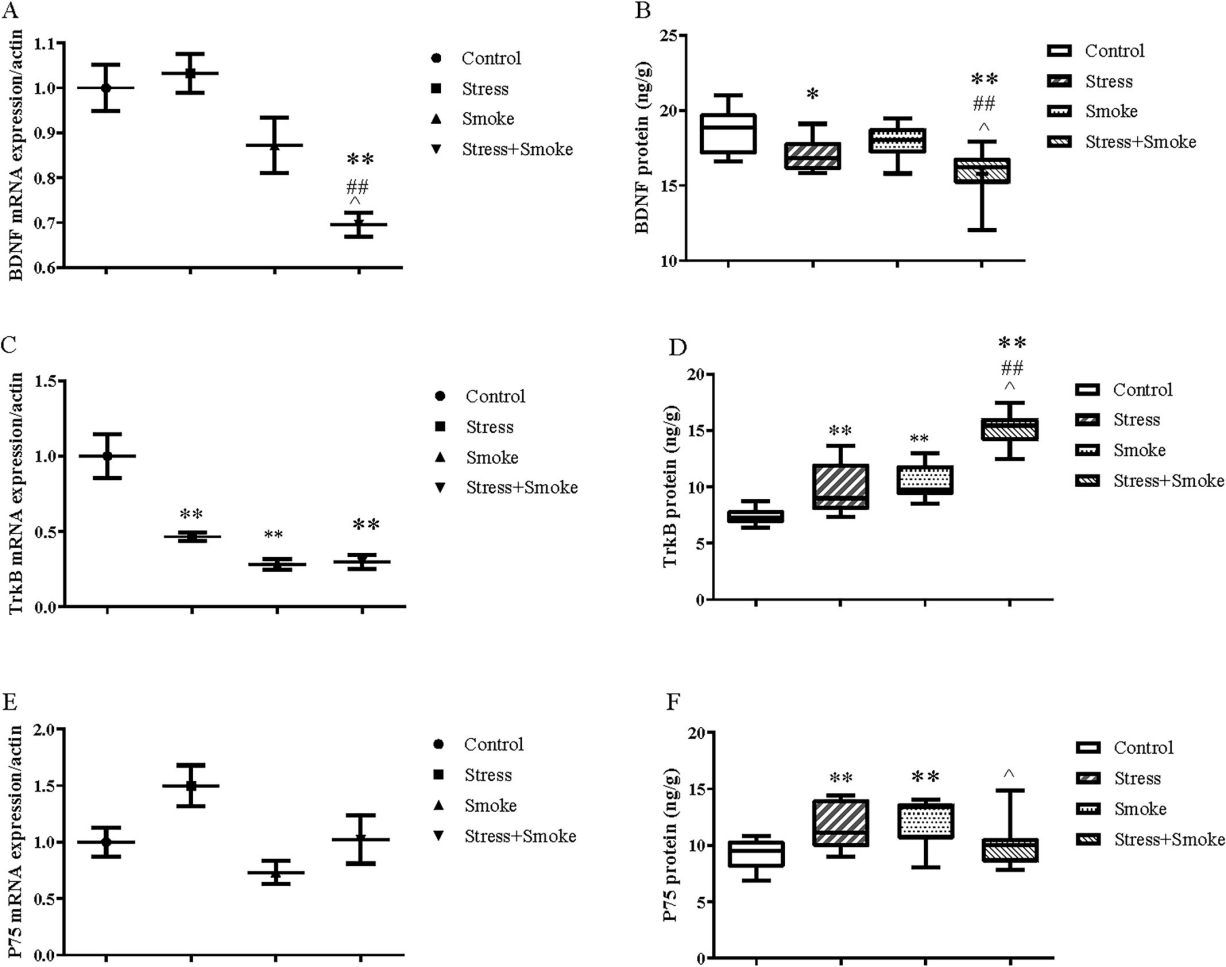
CUMS or smoke decreased protein concentration of BDNF, but increased TrkB and P75. However, both combinations caused more pronounced changes in BDNF and TrkB, but decreased P75 in the left lung. The mRNA expressions of BDNF, TrkB and P75 in the lung were measured by qPCR, while the concentrations were detected by ELISA kits. (A-B) mRNA and protein expression of BDNF. (C-D) mRNA and protein expression of TrkB. (E-F) mRNA and protein expression of P75. The data are expressed as mean ± SEM (n = 9–10). *P < 0.05, ^**^P < 0.01 versus control group; ^##^*P* < 0.01 versus stress group; ^^^*P* < 0.05 versus smoke group.

Two-way ANOVA indicated that CUMS significantly affected the mRNA expression of TrkB (F_1,33_ = 7.891, *P* < 0.01) and P75 (F_1,33_ = 4.460, *P* < 0.05) as well as the protein concentration of BDNF (F_1,33_ = 16.722, *P* < 0.01) and TrkB (F_1,33_ = 44.449, *P* < 0.01). Smoke exposure significantly affected both mRNA and protein expression of BDNF (mRNA, F_1,33_ = 16.508, *P* < 0.01; protein, F_1,33_ = 4.827, *P* < 0.05) and TrkB (mRNA, F_1,33_ = 23.210; protein, F_1,33_ = 65.466, both *P* < 0.01). Moreover, the combination significantly changed both mRNA and protein expression of TrkB (mRNA, F_1,33_ = 9.167, *P* < 0.01; protein, F_1,33_ = 4.829, *P* < 0.05), but only significant in protein expression of P75 (F_1,33_ = 12.454, *P* < 0.01). The *post hoc* test showed that CUMS, smoke or both combination significantly decreased the mRNA expression of TrkB (all in *P* < 0.01), while only the combination decreased the BDNF mRNA expression (*P* < 0.01). At the protein level, BDNF expression was reduced in stress (*P* < 0.05) and in combination (*P* < 0.01), but not in smoke group. However, CUMS or smoke largely increased TrkB (*P* < 0.01) and P75 (*P* < 0.01) concentrations compared to the control. Parallel to other pathological parameters, the decrease in both mRNA and protein expression of BDNF was much more pronounced in the combination group when compared to each single factor (mRNA: CUMS, *P* < 0.01; smoke, *P* < 0.05; protein: CUMS, *P* < 0.05; smoke, *P* < 0.01). As well, different from the effect of two single factor, the combination significantly increased protein concentration of TrkB (control and CUMS, *P* < 0.01; smoke, *P* < 0.05). However, the combination only decreased P75 (*P* < 0.05) when compared to the smoke (Fig 6).

### 3.7 The combination of CUMS and smoke decreased apoptotic factors in the left lung

**Fig 7:**
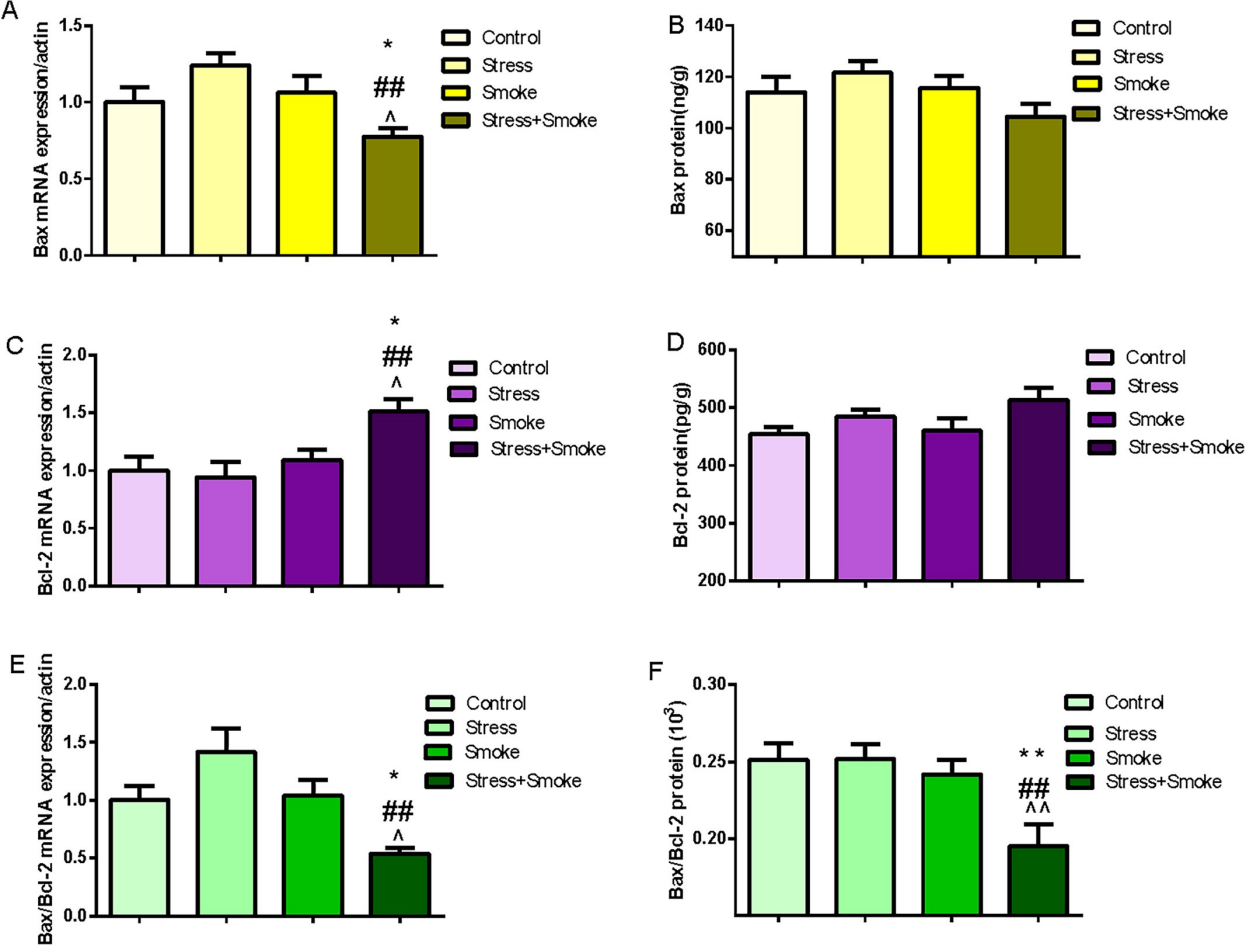
The combination of CUMS and smoke exposure significantly decreased the expression of pro-apoptosis factors and increase in anti-apoptosis factors when compared to stress or smoke group in the left lung. The mRNA expressions of Bax, Bcl-2 and Bax/Bcl-2 in the lung were measured by qPCR, while the concentrations were detected by ELISA kits. (A-B) mRNA and protein expression of Bax. (C-D) mRNA and protein expression of Bcl-2. (E-F) mRNA and protein expression of Bax/Bcl-2. The data are expressed as mean ± SEM (n = 9–10). *P < 0.05, ^**^P < 0.01 versus control group; ^*##*^*P* < 0.01 versus stress group; ^^^*P* < 0.05, ^^^^*P* < 0.01 versus smoke group.

Two-way ANOVA indicated that CUMS significantly affected Bcl-2 protein concentration (F_1,33_ = 6.118, *P* < 0.05) and Bax/Bcl-2 protein concentration ratio (F_1,33_ = 4.291, *P* < 0.05). Smoke exposure significantly influenced the Bax (F_1,33_ = 8.516, *P* < 0.05) and Bcl-2 (F_1,33_ = 4.371, *P* < 0.05) mRNA expression, Bax/Bcl-2 mRNA expression ratio (F_1,33_ = 8.712, *P* < 0.01) and the protein concentration of Bax/Bcl-2 (F_1,33_ = 8.865, *P* < 0.05). However, the *post hoc* test did not show any significant changes in these factors (Fig 6). Two-way ANOVA indicated that the interaction between CUMS and smoke significantly affected the mRNA expression of Bax (F_1,33_ = 5.698, *P* < 0.05), Bcl-2 (F_1,33_ = 8.031, *P* < 0.01), Bax/Bcl-2 (F_1,33_ = 9.085, *P* < 0.01) and the protein expression of Bax/Bcl-2 (F_1,33_ = 4.523, *P* < 0.05). The *post hoc* test showed that the combination significantly decreased Bax mRNA and Bax/Bcl-2 protein when compared to the control, CUMS or smoke group (*P* < 0.05; *P* < 0.01 and *P* < 0.05 respectively), while Bcl-2 mRNA (*P* < 0.05; *P* < 0.01; *P* < 0.05 respectively) were un-regulated when compared to the group with the single factor (Fig 7).

## 4. Discussion

The present study found that CUMS decreased sucrose consumption and increased immobility time, which validated the depression model. Then, both CUMS or smoke alone largely decreased body weight, mononuclear viability, TNF-α concentration in the left lung, BDNF and IL-10 in the amygdala, and BDNF, TrkB and IL-10 in the hippocampus, while increased corticosterone in the serum, HSP-90α, IL-1β, IL-2, IL-6, IL-8, IL-10 and IL-12 concentrations in the left lung, P75, IL-1β, IL-12 and TNF-α in the amygdala, and P75, IL-1β and TNF-α in the hippocampus. Most of these changes were exacerbated by the combination of CUMS and smoke. Thus, the present study for the first time demonstrated that synergic effects between CUMS and lung cancer inducer smoke exacerbated the depressive behaviors, tumorigenesis-related factor expression and brain and lung pathological changes. Several important points gained from these new findings are discussed below.

First, the present study showed that serum concentration of GC was significantly increased either in CUMS group or smoke group. Then the interaction between CUMS and smoke synergistically induced the greatest increase in this hormone. Many studies have already demonstrated that over-produced GC can directly cause anxious and depressive mood, activate glial cells to trigger neuroinflammation, induce neuronal apoptosis and neurodegeneration [61]. Therefore, the greatest increase in corticosterone level in the combination group may contribute to the most severe depression-like behaviors observed in the present study.

Furthermore, according to macrophage/T-lymphocyte theory of depression, excessive inflammatory response may contribute to the etiology of depression [62]. Our and others have reported that proinflammatory cytokines can stimulate the HPA axis to secret GC. Acute stress-induced GC usually suppresses immune function and exerts anti-inflammatory response [63]. However, chronic stress-induced GC can activate microglial M1 phenotype through CRF-receptor, which release proinflammatory factors and eventually cause neuroinflammation [64]. We and others have also reported that activated microglial M1 occurred in brains of depressed patients [65] or animal model of depression [66]. In the amygdala, CUMS or smoke significantly increased the concentrations of IL-1β, IL-12 and TNF-α, while decreased IL-10. Again, the changes in IL-10 and TNF-α were exacerbated in the combination group when compared to CUMS or smoke alone. We and others previously reported that increased corticosterone and neuroinflammatory stimuli could suppress astrocyte functions, such as reducing neurotrophin expression [42,67]. In consistent with neurotrophin hypothesis of depression, the present study found that at both mRNA and protein levels, CUMS or the combination significantly decreased the expression of BDNF and mRNA expression of TrkB, while a trend of decrease in protein expression of TrKB was found in the amygdala. By contrast, the expression of P75 receptor was increased. The binding of BDNF to its high affinity receptor TrkB could activate neuron survival associated signaling pathway PI3K/AKT [68]. When BDNF and TrkB expressions were decreased, BDNF binds to the low affinity receptor P75 to activate P75 pathway, which can trigger neuron apoptosis and induce depression [69]. For the hippocampus, the changes were similar to those in the amygdala. However, CUMS and smoke together did not further impair the function of BDNF system, which may result from that smoke alone did not completely cause the disruption of the BDNF system function.

It is well known that excessive release of GC, inflammation and deficient BDNF can all induce neuronal apoptosis. The present study found that CUMS significantly decreased the expression of anti-apoptosis factor bcl-2, while increased pro-apoptosis factor bax and bax/bcl-2 in the amygdala. The combination of CUMS and smoke exacerbated these changes. Thus, these findings in the brain demonstrated that CUMS can promote neuronal apoptosis and the occurrence of depression-like symptoms, while a synergistic effect between CUMS and lung cancer inducer smoke exposure can deteriorate depression-like symptoms and neuropathology.

Second, previous several studies have reported that increased GC can damage the circadian variation in cancer patients, which is similar to that observed in depression patients [70,71]. Indeed, GC can stimulate the tumor microenvironment [26] and promote tumor cell growth and invasion by inducing lymphocyte apoptosis, activating oncogenic viruses and inhibiting anti-tumor cellular responses [29,72]. Therefore, in the present study, the greatest increase in corticosterone in the combination group may contribute to greatest expression of lung cancer-related factors.

According to new hypothesis of cancers, inflammation can promote tumor growth, angiogenesis and invasion. Proinflammatory cytokines IL-1 and IL-6 act as tumor growth factors by combining specific receptors on the cell surface to induce the proliferation or prolongation of tumor cells. These proinflammatory cytokines can also mediate the expression of pro-metastatic genes, activate transcription factors and promote angiogenesis, such as VEGF, IL-2, IL-8 and IL-12 [73]. Interestingly, the present study showed that either CUMS or smoke increased the expression of IL-1, IL-2, IL-6, IL-8 and IL-12 in the lung even though no interaction between CUMS and smoke was found.

Furthermore, anti-inflammatory cytokine IL-10 can inhibit anti-cancer response by shifting CD4^+^ pattern to T regular pattern and promote Treg generation [74]. In the present study, CUMS or smoke increased the concentration of IL-10 in the lung, while the combination induced the most pronounced increase in the cytokine. This finding again indicates that two factors together promote lung cancer development.

Third, the decrease of mononuclear proliferation was reported in patients with depression or lung cancer [75,76]. The present study also found that mononuclear proliferation was decreased in either CUMS or smoke exposure group even though no interaction between CUMS and smoke was found. The present study at least provided evidence that CUMS or smoke, as promoter of lung cancer, significantly suppressed mononuclear proliferation.

On line with above findings in the immune system, the present study for the first time demonstrated that either CUMS or smoke exposure could significantly increase the expression of heat shock proteins 90 (HSP 90) α in the left lung, while two factors combination induced the most pronounced increase. The expression of HSP 90 is usually induced by heat shock or stress. There are two subtypes, HSP-90α and HSP-90β. Only HSP-90α could promote tumor development and progression, especially cancer metastasis [77,78]. Increased expression of HSP-90α protein was found in patients with lung cancer, while worse in patients with advanced lung cancer (stage Ⅲ) than patients with early-stage lung cancer (stage I) [79]. Since HSP-90α can activate matrix metalloproteinase-2 (MMP2) and then accelerate maturation of MMP2 to induce tumor angiogenesis, invasion and development [80,81], HSP-90α protein become a biomarker to detect lung cancer and chemotherapeutic efficacy [79,82]. Interestingly, the present study not only demonstrated CUMS or smoke alone can induce this lung cancer-related protein expression, and synergistic effect between two factors occurred, which further provide the evidence stress and lung cancer interaction to promote or worse lung cancer. In parallel with the increase in HSP-90α, the study found decreased TNF-α concentration in the left lung in the CUMS group or smoke group. The combination further decreased this cytokine when compared to the CUMS group. It is well known that TNF-α is a pro-inflammatory cytokine, which displays both apoptotic and anti-apoptotic properties in cancer cells, depending on the nature of the stimulus and active status of certain signaling pathways. The anti-apoptotic effect of TNF-α is through up-regulation of NF-κB activity. By contrast, the inhibition of TNF-α-mediated NF-κB activity represents an apoptosis effect [83]. Importantly, the present study not only showed CUMS or smoke alone can inhibit lung cell apoptosis, but also demonstrated the synergistic effect between CUMS and smoke, which further suggests that CUMS can promote the development of lung cancer.

Furthermore, this study found that the expression of other lung cancer-related factors CDK1, CDC20, P38α and CUEDC were all significantly increased in the left lung of stress+smoke mice when compared to the each single group. Furthermore, CDK1 and CDC20 protein concentrations also showed similar changes. However, ROS mRNA expression in stress+smoke group was increased when compared to the control and stress alone group, and Gankyrin mRNA expression was increased when compared to the control group. These results strongly suggested that CUMS and smoke combination was the worst trigger for lung cancer development.

Fourth, the dysfunction of neurotrophins, including BDNF and its receptors TrkB/P75 play an essential role in both mental diseases and lung cancer [84,85]. BDNF deficiency was reported in depressed patients or models [86]. Interestingly, recent evidence showed that serum BDNF level was down-regulated in the patients with advanced small cell lung cancer [87]. In the present study, the protein concentration of BDNF in the lung was decreased in CUMS group compared to control group, while the combination of CUMS and smoke exacerbate the decrease when compared to CUMS or smoke alone. The protein expression of TrkB in the lung was significantly increased in CUMS or smoke group, while the most increase in the TrkB receptor was found in stress+smoke group. The activation of TrkB could enhance the activity of STAT3 and its downstream PI3K/AKT signaling pathway, which eventually promote lung cancer cells proliferation [85]. Furthermore, the protein concentration of P75 in the lung, another BDNF receptor, was also significantly increased in CUMS or smoke group, whereas was significantly decreased in stress+smoke group compared to smoke group. It is known that P75 can inhibit lung cell proliferation via the up-regulation of p53 and UNC5D to induce expression of pro-apoptotic target genes [88]. Therefore, we supposed that the elevated P75 level in CUMS or smoke group may indicate an effect of inhibiting lung cell proliferation as a complement. But decreased P75 may indicate that the inhibition was damaged upon the combination. These data at least demonstrated that the dysfunction of BNDF system in the lung after exposing to CUMS, smoke or their combination significantly increased the risk of lung cancer development.

Fifth, opposite to neurons, excessive corticosterone secretion, inflammatory response, or neurotrophin dysfunction can all promote lung cancer cell proliferation [89–91]. In the present study, the combination of CUMS and smoke significantly decreased Bax/Bcl-2 when compared to each group with the single factor in the lung. Therefore, the greatest increase in corticosterone and inflammation, and the worst neurotrophin dysfunction in the combination group may contribute to greatest expression of lung cancer-related factors in the lung.

**Fig 8:**
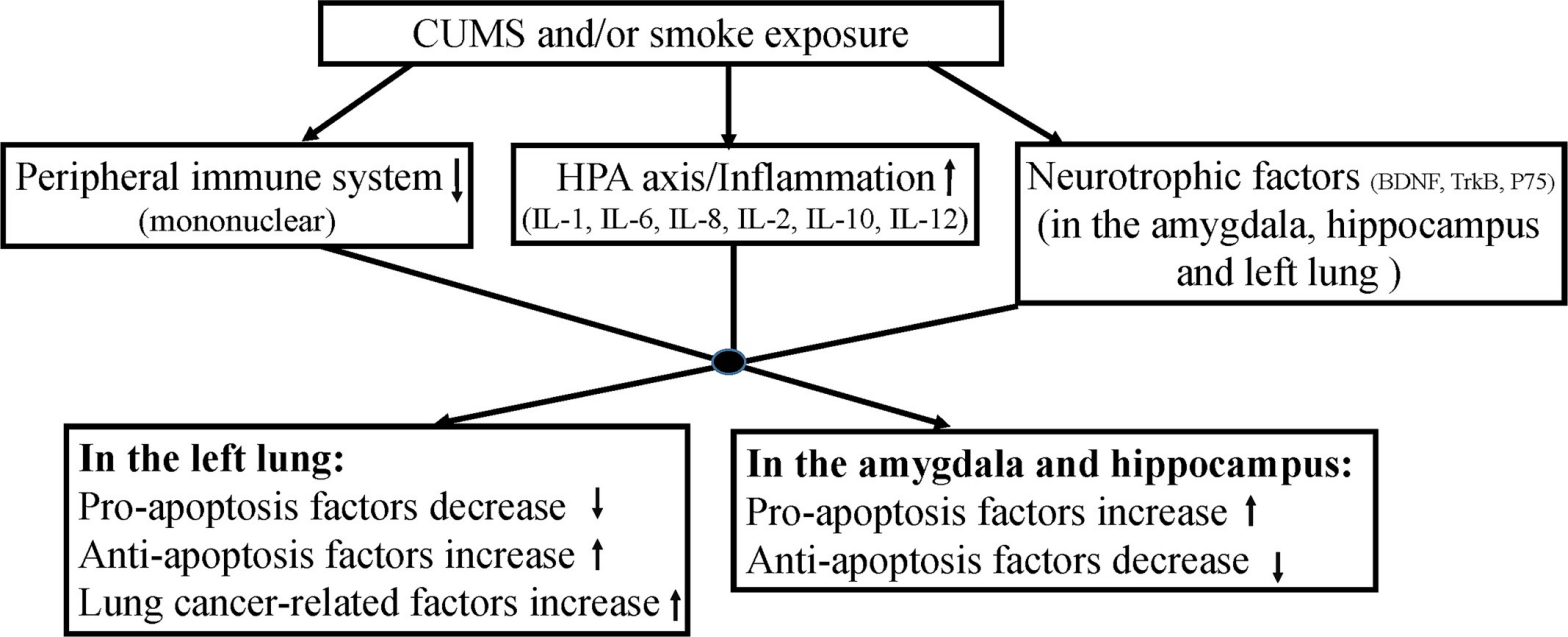
The summary of schematic diagram of the study.

In summary, this study for the first time provided psychoneuroimmunological evidence to support that CUMS could activate the HPA axis, elevate inflammation in the amygdala and peripheral, inhibit peripheral immune and damage lung and amygdale BDNF system (Fig 8). Moreover, the combination of CUMS and smoke together in lung cancer vulnerable mice was more likely to promote the development of lung cancer and worse depression-like behaviors and pathological changes. The possible common biological mediators between depression and lung tumorigenesis are abnormalities in GC secretion, inflammatory response and BDNF function.

Even though, several parameters directly related to lung cancer development were increased by CUMS and smoke combination, no solid tumors emerged in this group after 8 weeks of two factor administration. The result may suggest that the effect of longer duration of CUMS plus smoke exposure on lung cancer growth should be studied in the future.

However, it is unlike that 8 weeks is long enough to grow up a visible tumor from scratch. Since clinic investigations have reported that chronic stress and smoke for several or many years are coincident with cancer onset or deterioration, it is reasonable that lung cancer did not obviously appear after two months stress plus smoke. Therefore, these results at least clearly showed that CUMS and smoke combination indeed increased the risk of lung cancer.

## Supporting information

S1 Fig The photo of the apparatus used in the smoke exposure. (TIF)

S2 Fig The mRNA expression of caspase3 in the amygdala. (TIF)

S3 Fig HE staining of lung tissue (200×). (TIF)

S1 Table CUMS procedure. (PDF)

S1 File WB image. (PDF)

## Abbreviations

CUMS: Chronic Unpredictable Mild Stress
BDNF: Brain-Derived Neurotrophic Factor
(IL)-1β: Interleukin-1β
GC: Glucocorticoid
HPA axis: Hypothalamic-Pituitary-Adrenal Axis
PLA2: Phospholipase A2
PGE2: Prostaglandin E2
IFN-γ: Interferon-γ
TNF-α: Tumor Necrosis Factor-α
GR: Glucocorticoid Receptor
HSP 90: Heat Shock Proteins 90
MMP2: Matrix Metalloproteinase-2
