# Supplementary figures and images for: The combination of chronic stress and smoke exacerbated depression-like changes and lung cancer factor expression in A/J mice: Involve inflammation and BDNF dysfunction
Source: PLoS One. 2022 Nov 23;17(11):e0277945. doi: 10.1371/journal.pone.0277945 (PMC9683596; doi:10.1371/journal.pone.0277945)

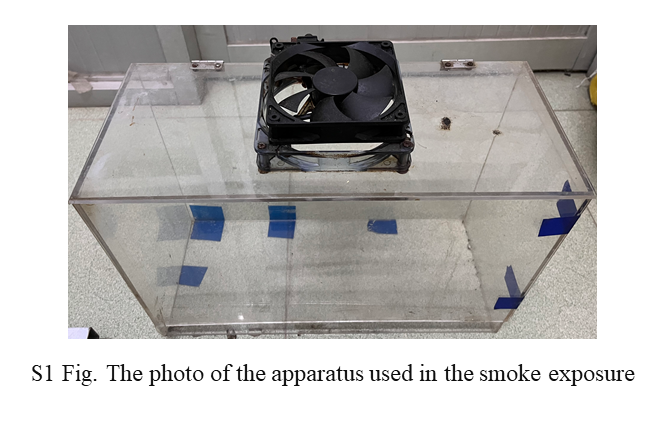

Supplement: S1 Fig — (TIF) [file pone.0277945.s001.tif]

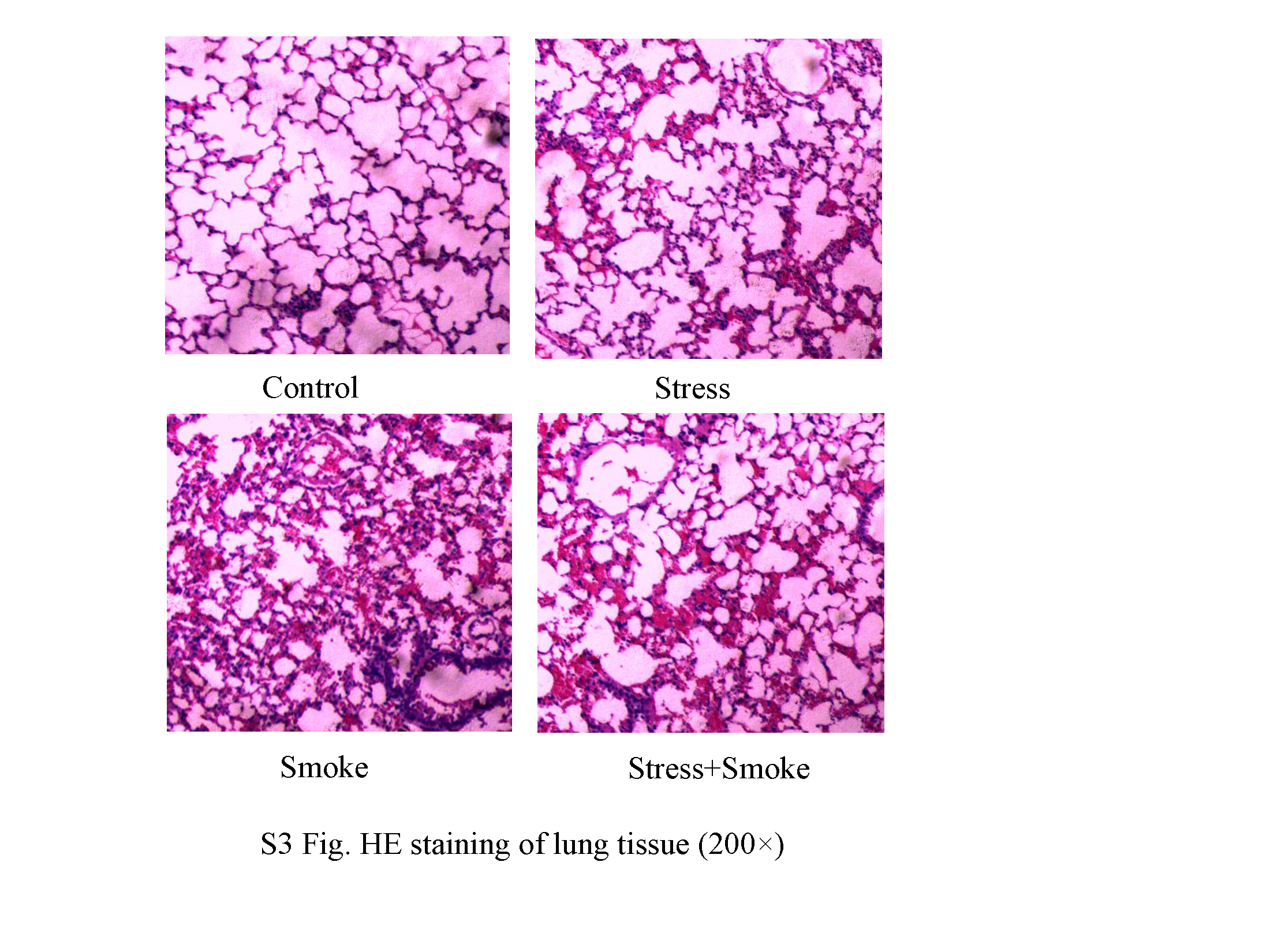

Supplement: S3 Fig — (TIF) [file pone.0277945.s003.tif]
