# Supplementary material for: The combination of chronic stress and smoke exacerbated depression-like changes and lung cancer factor expression in A/J mice: Involve inflammation and BDNF dysfunction
Source: PLoS One. 2022 Nov 23;17(11):e0277945. doi: 10.1371/journal.pone.0277945 (PMC9683596; doi:10.1371/journal.pone.0277945)
Supplement: S1 Table — (PDF) [file pone.0277945.s004.pdf]

S1 Table. CUMS procedure

Stressors	Application day (D)
cold exposure (4℃) for 1 h	D2，10，18，23，29，33，40，43，49，52
forced swimming at 18℃ for 10 min	D3，12，22，26，32，39，44，48，53
restraint for 2 h	D1，7，14，19，27，35，38，43，47，55
overnight illumination	D1，12，19，23，31，39，42，44，50
shaking at 200 rpm for 2 h	D5，8，18，22，28，33，45，54
isolation overnight	D5，10，15，25，29，34，42，50
cage tilting at 45° for overnight	D7，17，24，32，41，46，51，55
stroboscopic illumination for overnight	D2，8，13，21，27，30，36，40，56
crowded space for overnight	D6，9，16，24，30，38，45，53
odor overnight	D3，9，14，20，26，34，46，54
food and water deprivation for overnight	D4，11，15，20，28，37，41，49，56
light on and off every 3 h for 24 h	D6，13，17，25，35，37，47，51
soiled cage overnight	D4，11，16，21，31，36，48，52
