# Supplementary material for: The combination of chronic stress and smoke exacerbated depression-like changes and lung cancer factor expression in A/J mice: Involve inflammation and BDNF dysfunction
Source: PLoS One. 2022 Nov 23;17(11):e0277945. doi: 10.1371/journal.pone.0277945 (PMC9683596; doi:10.1371/journal.pone.0277945)
Supplement: S1 File — (PDF) [file pone.0277945.s005.pdf]

WB image:

TrkB ①:

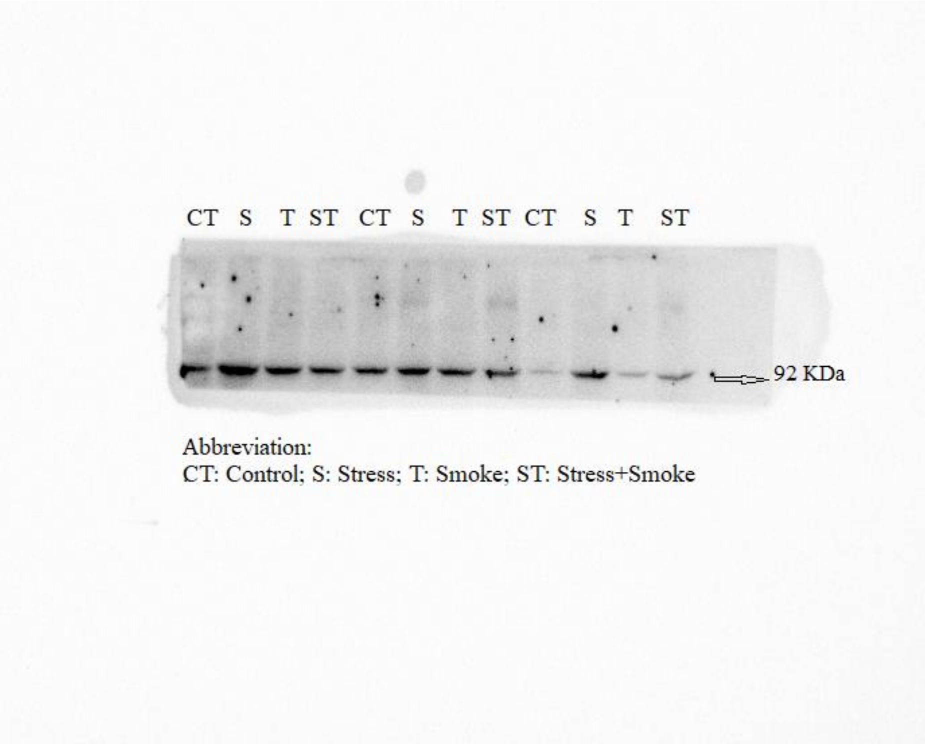

β-actin ①:

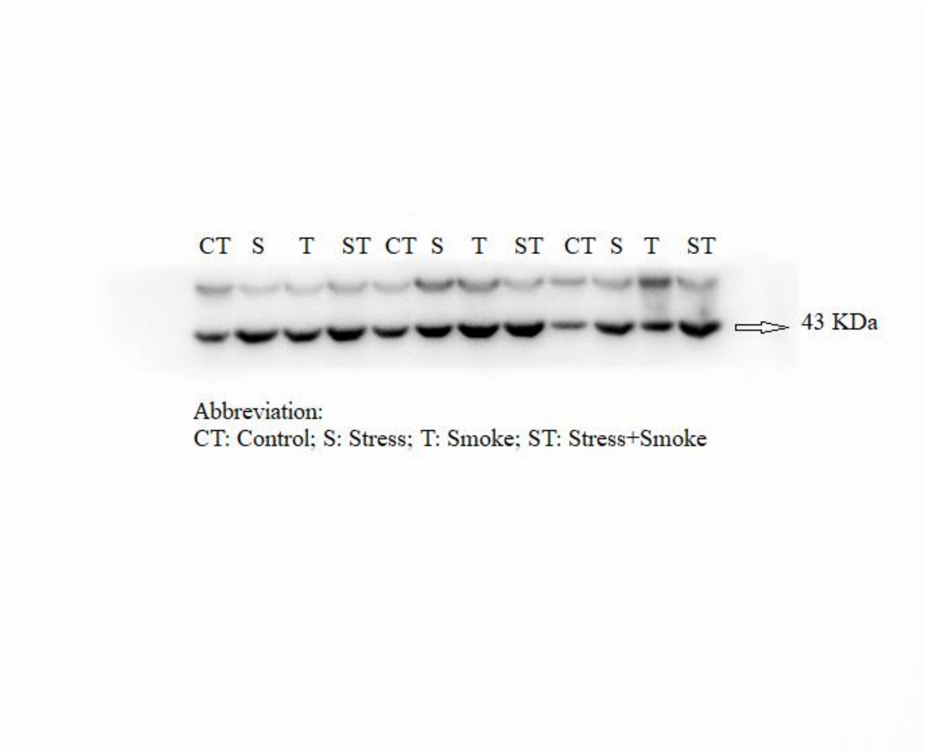

TrkB ②:

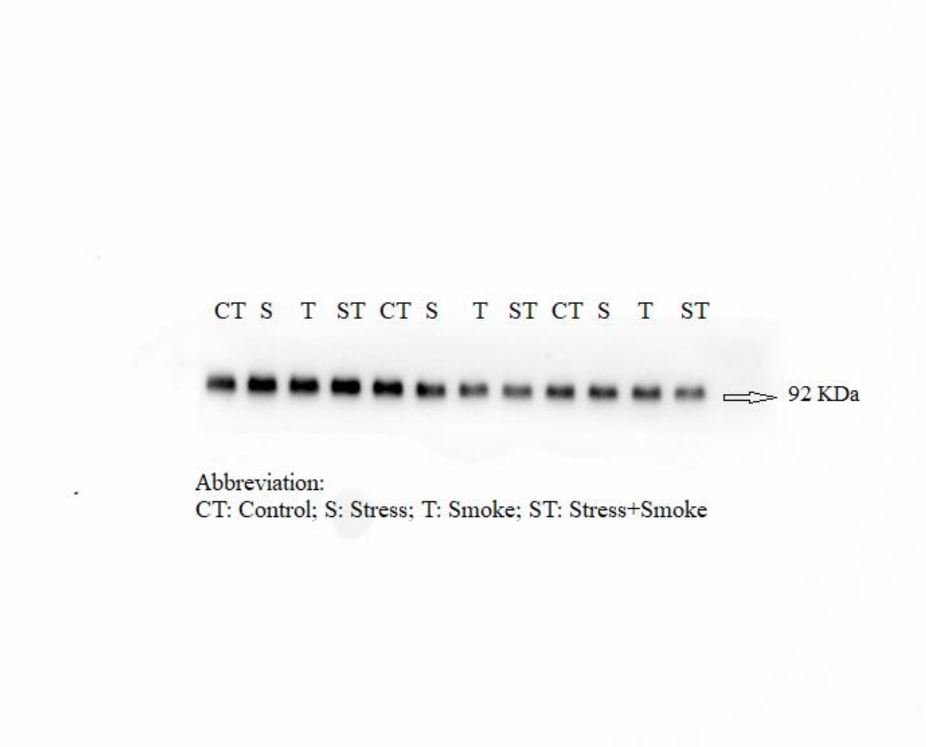

β-actin ②:

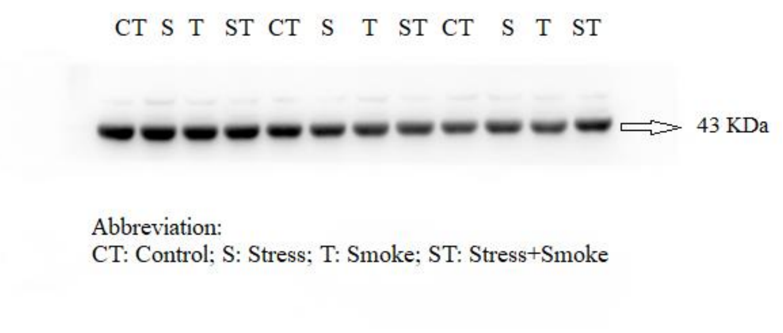

P75 ①:

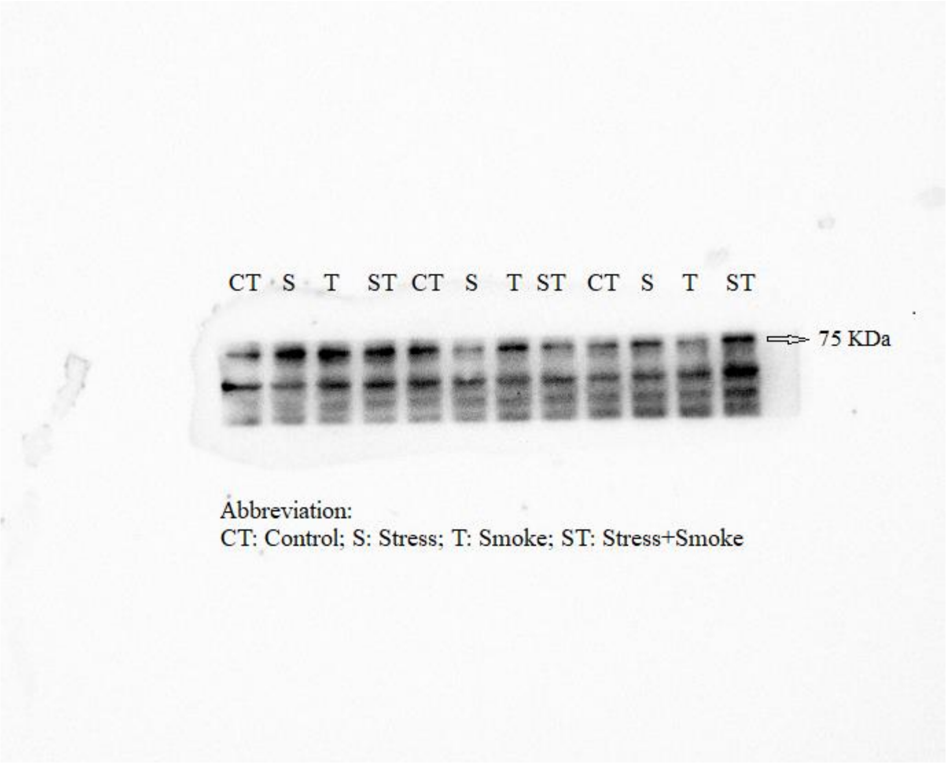

β-actin ①:

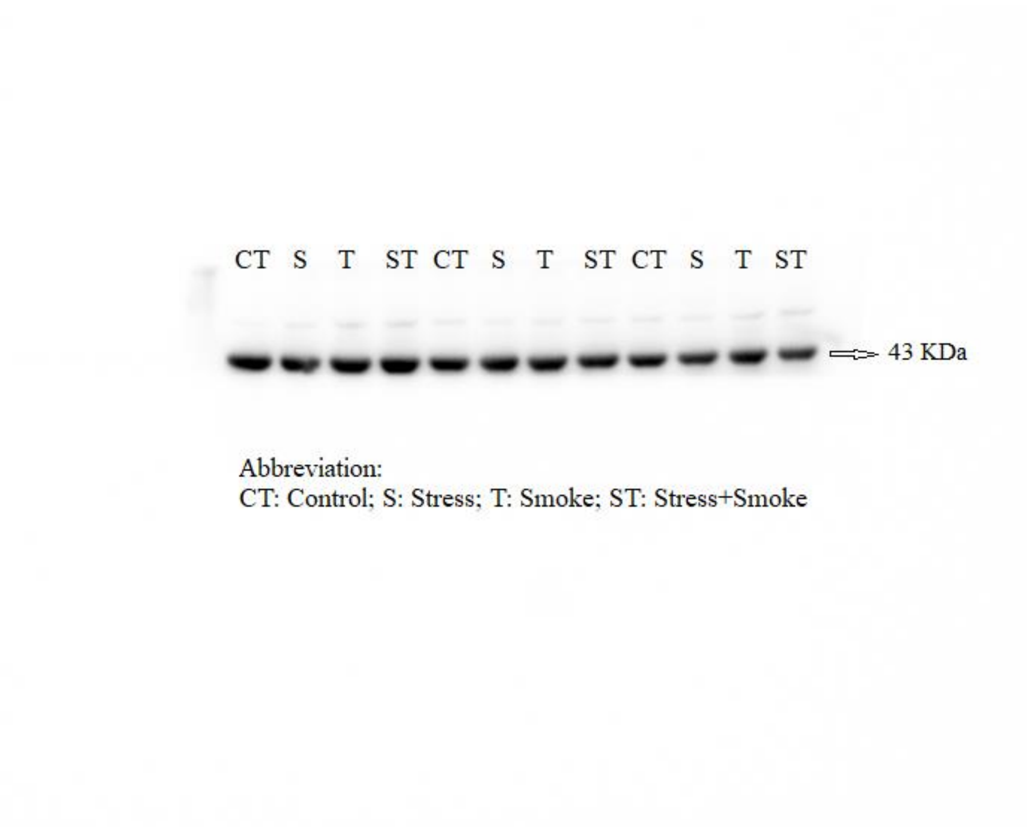

P75 ②:

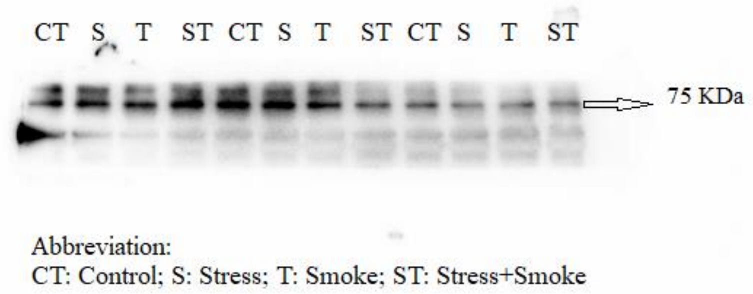

β-actin ②:

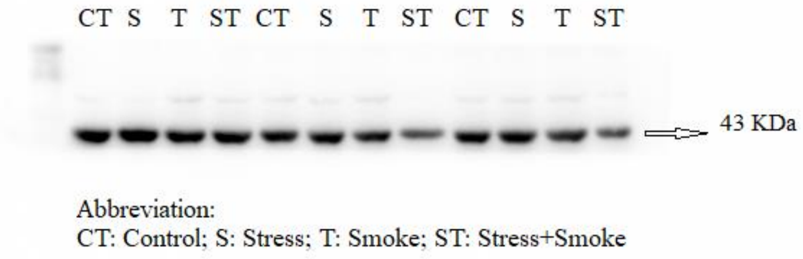

P75 ③：

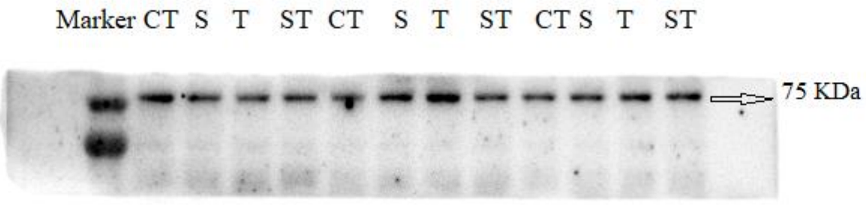

Abbreviation:
CT: Control; S: Stress; T: Smoke; ST: Stress+Smoke

β-actin ③:

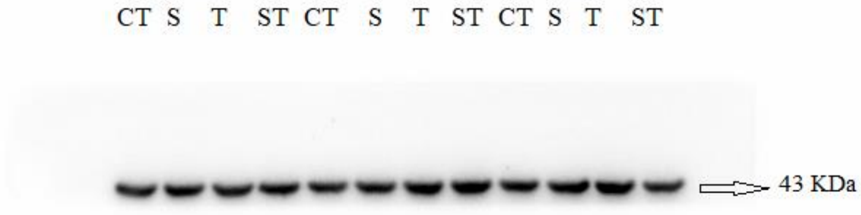

Abbreviation:
CT: Control; S: Stress; T: Smoke; ST: Stress+Smoke
